# Strontium-doped hydroxyapatite polysaccharide materials effect on ectopic bone formation

**DOI:** 10.1371/journal.pone.0184663

**Published:** 2017-09-14

**Authors:** C. Ehret, R. Aid-Launais, T. Sagardoy, R. Siadous, R. Bareille, S. Rey, S. Pechev, L. Etienne, J. Kalisky, E. de Mones, D. Letourneur, J. Amedee Vilamitjana

**Affiliations:** 1 Inserm U1026, University of Bordeaux, Tissue Bioengineering, U1026, Bordeaux, France; 2 Inserm U1148, LVTS, X. Bichat Hospital, University Paris Diderot F-75018 Paris, Institut Galilée, University Paris 13, Villetaneuse, France; 3 ICMCB, Bordeaux University, Bordeaux, France; 4 CHU Bordeaux, Oral and Maxillo-Facial Department, Bordeaux, France; Universite de Technologie de Compiegne, FRANCE

## Abstract

Previous studies performed using polysaccharide-based matrices supplemented with hydroxyapatite (HA) particles showed their ability to form in subcutaneous and intramuscular sites a mineralized and osteoid tissue. Our objectives are to optimize the HA content in the matrix and to test the combination of HA with strontium (Sr-HA) to increase the matrix bioactivity. First, non-doped Sr-HA powders were combined to the matrix at three different ratios and were implanted subcutaneously for 2 and 4 weeks. Interestingly, matrices showed radiolucent properties before implantation. Quantitative analysis of micro-CT data evidenced a significant increase of mineralized tissue formed ectopically with time of implantation and allowed us to select the best ratio of HA to polysaccharides of 30% (w/w). Then, two Sr-substitution of 8% and 50% were incorporated in the HA powders (8Sr-HA and 50Sr-HA). Both Sr-HA were chemically characterized and dispersed in matrices. *In vitro* studies performed with human mesenchymal stem cells (MSCs) demonstrated the absence of cytotoxicity of the Sr-doped matrices whatever the amount of incorporated Sr. They also supported osteoblastic differentiation and activated the expression of one late osteoblastic marker involved in the mineralization process *i*.*e*. osteopontin. *In vivo*, subcutaneous implantation of these Sr-doped matrices induced osteoid tissue and blood vessels formation.

## Introduction

For patients encountering a surgical bone reconstruction, new developments in orthopaedic and cranio-maxillofacial surgery are expected, especially for non-union fractures, large bone defects, bone physiopathologies and bone reconstruction following tumor resection [1]. In such clinical context, the reconstruction of large volume defects remains challenging because of a lack of vascularization of the newly formed tissue. Clinical alternatives to autografts and allografts [2] include i) the use of biomaterials [3] such as bioceramics, metallic implants (*e*.*g*. titanium alloys), natural or synthetic polymers, ii) the production of tissue-engineered constructs that combine scaffolds with mesenchymal stems cells [4, 5] and endothelial progenitor cells [6], growth factors, especially osteoinductive factors, or angiogenic growth factors [6]. However, tissue-engineering strategies are facing the difficulty to obtain reproducible and sufficient autologous cells from non-healthy patients with an osteogenic capacity. Tissue-engineered constructs are also labor intensive to produce with low throughput, and these advanced cell therapies are associated with stringent regulatory hurdles. Additionally, when considering an oncological context for bone reconstruction, autologous undifferentiated cells with capacity for self-renewal have a potential for tumor formation. While the use of Bone Morphogenic Proteins (BMPs) has been approved for specific bone regeneration applications, their use is contraindicated in an oncological context, due to concerns that this anabolic growth factor may contribute to tumor cell proliferation [7]. Consequently, the development of cell-free and growth factor-free approaches for the replacement of diseased or damaged bone tissue is expected for such specific clinical needs.

Based on the nature and structure of bone tissue as a composite material, composed of nano-, micro- and macro-scale elements, natural polymers have an advantageous role in the production of biocomposite scaffolds, made of an organic polymeric materials and an inorganic product [8]. Among these polymers, collagen, chitosan, dextran, alginate are the typical biomaterials used to produce natural-based biocomposites [5, 8]. Some of them are injectable and have intrinsic properties for preclinical applications [9]. These nano-composites scaffolds have shown promise as a synthetic bone graft substitute for stimulating osteoconductive properties of the materials, with improved architecture and mechanical properties [10]. Since natural bone is composed of nano-sized hydroxyapatite crystals with needle-like morphology, numerous papers described the synthesis of hydroxyapatite with a bone-like nanometric structure for tissue engineering applications [11, 12]. Particles of hydroxyapatite (HA) are synthesized using different methods and temperature (precipitation, wet mechanochemical method, calcination process) [13, 14] and numerous synthesized routes lead to different productions of HA, exhibiting different shapes, sizes (from 50 nm to few micrometers as nanoparticle aggregates) different biological characteristics and bioactive properties [11]. The mode in which aggregation takes place plays an important role in their biological properties. Additionally, the amount of HA within the polymer and their mode of dispersion can modify the bioactive properties of the composite scaffold [15].

Besides the nanophase of HA, as previously described in the literature [6], the major calcium phosphate component of bone tissue is not an homogenous material, and bone incorporates also other elements (*i*.*e*. Na^+^, Mg^2+^, or Sr^2+^) in the form of trace elements [16]. These trace elements have been found to play vital function in growth and bone repair [17]. They can control the degradation, increase the mechanical strength of the materials and positively regulate their bioactive properties such as osteoconduction [17]. One of the cations that can substitute for calcium in the structure of HA, the Strontium (Sr), has drawn increasing interest [18] because of its beneficial effect on bone formation and its prevention of bone resorption [16, 19]. Its clinical application for osteoporosis treatment [20, 21] has been reported as well as an enhanced osteoporotic bone regeneration by strontium-substituted materials [22, 23]. *In vitro* and *in vivo*, the effect of Sr seems to be connected to the ratio of Sr-substituted within the HA [24–26]. Numerous *in vitro* studies have described that Sr-doping in HA facilitates proliferation and differentiation of osteoblasts [27–30] by activating calcium-sensing receptor, which stimulates both osteogenesis and angiogenesis [31, 32]. A low dose of Sr has previously shown to increase bone mineralization while a high concentration could have a negative effect [26, 33, 34].

Our previous work using macroporous polysaccharide-based scaffolds composed of pullulan-dextran combined with 14% of nanocrystalline hydroxyapatite particles, showed their ability to form, ectopically, a mineralized and an osteoid tissue in two ectopic sites (subcutaneously in mice and intramuscularly in sheep) [35]. Therefore, hypothesis of this study was to test the combination of HA and strontium at a specific ratio, that should improve the bioactivity of this natural polysaccharide-based matrix, especially angiogenesis.

In this study, particles of hydroxyapatite with different ratios of Sr-substitution (8% and 50%) were synthesized and characterized by X-ray diffraction (XRD), Fourier transform Infrared Spectroscopy (FTIR), Transmission Electron Microscopy (TEM) and Inductively coupled plasma optical emission spectrometry (ICP-OES) for the determination of the Sr and calcium (Ca) content. The non-substituted and substituted hydroxyapatites were then combined to the polysaccharide sat different ratios. Matrices doped or not with strontium were further studied by environmental scanning electron microscopy (ESEM), by ICP-OES. *In vitro* cellular behaviour of human mesenchymal stem cells, and ectopic mineralization and bone formation after their subcutaneous implantation in mice were then investigated.

## Materials and methods

### Synthesis of hydroxyapatite particles

HA particles were synthesized by wet chemical precipitation [14] with some modifications. Briefly, HA particles were synthesized using 50 mL of 1.08 M Ca(NO_3_)_2_, 4H_2_O solution and 50 mL of 0.65 M (NH_4_)_2_HPO_4_ solution, heated at 90°C. The pH of solution was adjusted to 10 with NH_4_OH, added dropwise under stirring. The precipitate was maintained for 5 h at 90°C under stirring, and then washed 4 times with CO^2^-free distilled water. Strontium substituted hydroxyapatite (Sr-HA) was obtained following the same procedure by adding Sr^2+^ions into the Ca2+ solution, before adjusting the pH to 10 with NH_4_OH. For this purpose, the appropriate amounts of Sr (NO_3_)_2_ and Ca (NO_3_)_2_·4H_2_O were dissolved in order to obtain 8% or 50% (molar ratio) of Ca^2+^ substituted by Sr^2+^, 8Sr-HA and 50Sr-HA, respectively. The concentrations of the different HA solutions were determined after freeze-drying of one sample for each group.

### Physicochemical and morphological characterization of hydroxyapatite particles

Powder X-ray diffraction (XRD) patterns were collected on a PANalitycalX'pert PRO MPD diffractometer in Bragg-Brentano θ-θ geometry equipped with a secondary monochromator and X'Celerator multi-strip detector. The Cu-Kα radiation was generated at 45 KV and 40 mA (l = 0.15418 nm). Each measurement was made within an angular range of 2θ = 8–80°. The samples were put on Zero Background sample holder made of obliquely cut silicon crystal.

Infrared spectroscopy (FTIR) was performed by using a BRUKER Equinox 55(Bruker GmbH, Germany) with a spectral resolution of 4 cm^-1^. Samples were prepared by mixing 2 mg of ground sample with 100 mg of anhydrous KBr. Sample spectra were recorded immediately after the background acquisition. Automated spectra acquisition was controlled by a small macro-written in the OPUS Macro Language Software of the spectrometer.

The particle sizes and their morphology were examined by Transmission Electron Microscopy (TEM) (HITACHI H7650). Dried samples were dispersed on lacy carbon Cu grids by contact with the grids and subsequent gentle shaking. The particle sizes of the hydroxyapatite aqueous solutions were also analysed by dynamic light scattering (DLS) with the Malvern Zetasizer equipment. For all HA samples, the particles were first suspended in a borate buffer 20 mM pH 8.5 and then subjected to size measurements.

Inductively coupled plasma optical emission spectrometry (ICP-OES) was applied to determine the content of calcium and strontium in the mineral phase of non-substituted HA (HA) and Sr-substituted hydroxyapatite (Sr-HA). Powders were preliminary dissolved in 5 mL of HCl 2–3 days before ICP analysis (ICP-OES 720-ES, Varian). The resulting solutions were analysed for Ca and Sr concentrations with ICP-OES instrument calibrated with standard solutions of known concentrations of these elements. Based on the measured concentrations, the absolute amount of each individual element within HA powders was calculated and expressed in μg of element / mg of powder.

### Synthesis of the macroporous HA-matrices and Sr-HA-matrices and characterization

Polysaccharide-based matrices were synthesized using a blend of pullulan/dextran 75:25 (pullulan, MW 200,000, Hayashibara Inc.; dextran, MW 500,000, Sigma) prepared by dissolving 9 g of pullulan and 3 g of dextran into 40 mL of distilled water containing 14 g of NaCl [36, 37]. Macroporous scaffolds combined with hydroxyapatite particles (Matrix-HA) were produced as previously described [35]. Here, distilled water was replaced by the hydroxyapatite suspensions at a concentration of 6.7 ± 1.7 g/mL, doped or not with strontium (HA, 8Sr-HA and 50Sr-HA) at three different ratios of dispersion (D1, D2 and D3). Our previous work was performed with non-doped HA dispersed at D1 leading to 14% (w/w) of HA amount [35]. Chemical cross-linking was carried out using trisodium trimetaphosphate. After incubation at 50°C for 15 min, resulting scaffolds were cut into the desired shape (6 mm diameter, 1.5 mm thickness), soaked in PBS0.1M pH 7, then washed extensively with a 0.025% NaCl solution. After freeze-drying, scaffolds were stored at room temperature until use. Matrices containing HA particles are named Matrix-HA (for non-doped HA), Matrix-8Sr-HA (8% of Sr-substitution) and Matrix-50Sr-HA (50% of Sr-substitution). The exact amount of mineral phase within the matrices produced with the three ratios of HA / polysaccharides, *i*.*e*. D1, D2 and D3, was calculated and reported in Table 1. To obtain these quantitative data, polysaccharide-based scaffolds (Matrix-HA and Matrix-Sr-HA) were degraded *in vitro* with a combination of pullulanase (from *Bacillus subtilis*, 44.4 U/mL; Sigma-Aldrich) and dextranase (from *Chaetomium erraticum*, 5.3 U/mL; Sigma-Aldrich) that do not degrade hydroxyapatite. The enzymatic solution was added to the scaffolds for 1 hour at 37°C. The hydroxyapatite that remained at the bottom of the tube was then rinsed in osmosed water and finally dried at 50°C in vacuum drying oven overnight. Finally, the weight was determined.

**Table 1:** Hydroxyapatite (substituted or not with Sr) content in the different matrices produced at the three conditions of dispersion (D1, D2 and D3).

	Hydroxyapatite content in the matrix (%)		
HA powder samples used for matrix production	Dispersion 1 (D1)	Dispersion 2 (D2)	Dispersion 3 (D3)
**HA**	14.2 +/- 2.76	30.5 +/- 3.21	65.6 +/- 1.42
**8Sr-HA**	13.1 +/- 1.47	32.8 +/- 0.96	62.5 +/- 2.36
**50Sr-HA**	14.8 +/- 2.53	28.9+/- 2.28	61.4 +/- 1.15

Hydrated scaffolds were observed using environmental scanning electron microscopy (ESEM) with a FEI QUANTA 200 at an accelerating voltage of 15 keV and at a pressure of 3.5 Torr.

Inductively coupled plasma optical emission spectrometry (ICP-OES) was applied to determine the content of strontium incorporated in the composite scaffolds with ICP-OES analysis instruments calibrated with standard solutions of known concentrations of the element. Matrices were preliminary dissolved in 0.5 mL HNO_3_ and 0.5 mL HCl for 2–3 days before ICP analysis (ICP-OES 720-ES, Varian). Based on the measured concentrations, the amount of Sr per matrix was calculated and expressed in ng / Matrix.

### Cell behavior of human mesenchymal stem cells

Human mesenchymal stem cells (MSCs) were isolated as previously described [38]. Briefly, human bone marrow was aspirated from the femoral diaphysis or iliac bone after obtaining consent from patients (40–70 year-old) undergoing hip prosthesis surgery after trauma. The human bone marrow was then sequentially filtered with syringes fitted with 16-, 18-, and 21-gauge needles. Culture medium was Dulbecco’s Modified Eagle’s Medium (DMEM, Gibco) supplemented with 10% (v/v) Foetal Bovine Serum (FBS, Gibco) and cells were maintained in this basal medium for *in vitro* experiments with the different scaffolds.

Disks (6 mm diameter x 1.5mm thickness) of matrices were seeded with a density of 2.5 x 10^5^ MSCs per scaffold (Matrix-HA, Matrix-8Sr-HA, Matrix-50Sr-HA) in DMEM containing 10% (v/v) FCS without osteogenic additives. Cultures were incubated at 37°C in a humidified atmosphere for 3 and 7 days. The culture medium was replenished every 2 days. The viability of cells cultured within the scaffolds was evaluated at day 3 and day 7 of culture using a L3224 LIVE/DEAD^®^ viability/cytotoxicity kit according to the manufacturer’s protocol (Molecular Probes). The 3D cultured cells were stained with calcein AM and ethidium homodimer-1. Membrane-permeant calcein AM is cleaved by esterase in live cells to yield cytoplasmic green fluorescence and membrane-impermeant ethidium homodimer-1 labels membrane-compromised cells with red fluorescence. Images were recorded with a Leica TCS SP5 confocal microscope (Leica Microsystems, Wetzlar, Germany).

### RNA isolation and relative gene expression by quantitative real time PCR analysis

Cells were seeded with a density of 2.5 x 10^5^ MSCs per scaffold (Matrix-HA, Matrix-8Sr-HA, Matrix-50Sr-HA), produced at the dispersion D2 of hydroxyapatite. Total RNA was extracted from cells after 1, 3 and 7 days of culture, using the Nucleospin^®^ RNA kit (Macherey-Nagel, Düren, Germany). Briefly, scaffolds were rinsed with PBS 0.1 M pH 7, dried and frozen at—80°C for 5 hours before RNA extraction according to the manufacturer instructions. 500 ng of RNA were used as template for single-strand cDNA synthesis with the Maxima system (Thermo Fisher Scientific, USA) in a 20 μl final volume. Quantitative PCR (QPCR) amplification was performed using the TAKYON (2 ´ iQ 50 mMKCl, 20 mMTris–HCl, pH 8.4, 0.2 mM each dNTP, 25 U/mL iTaq DNA polymerase, 3 mM MgCl2, SYBR Green I and 10 nM fluorescein, stabilized in sterile distilled water). Primers of the ubiquitary ribosomic protein P0 (forward 5’- CCT CGT GGA AAG TGA CAT CGT -3’, reverse 5’- ATC TGC TTG GAG CCC ACA TT—3’), Runx2 (forward 5’-TCA CCT TGA CCA TAA CCG TCT-3’, reverse 5’- CGG GAC ACC TAC TCT CAT ACT-3’), osteopontin (OPN) (forward 5’- GCC GAG GTG ATA GTG TGG TT—3’, reverse 5’- AAC GGG GAT GGC CTT GTA TG– 3’) were used at a final concentration of 200 nM. Data were analyzed using iCycler IQ software and compared by the ΔΔCT method. Q-PCR was performed in duplicate for PCR validation. Data were normalized to P0 expression for each condition. Runx2 and OPN gene expression are expressed in relative expression compared to day 1 for each condition and also compare to Matrix-HA for each time of culture.

### Implantation of the HA-matrices and Sr-HA- matrices subcutaneously in mice

Animal experiments were performed in accordance with the ‘‘Principles of Laboratory Animal Care” recommended by the National Society for Biomedical Research in France. Interventions were carried out in an accredited animal facility (authorization nu A33-063-917) at the University of Bordeaux, under authorization nu 4375–2016030408537165 v4 of the French Ministry of Agriculture and were approved by the Animal Ethic Committee. Scaffolds (6 mm diameter, 1.5 mm thickness) of i) Matrix-HA containing different ratios of HA (D1, D2 and D3) or ii) Matrix-HA, Matrix-8Sr-HA, Matrix-50Sr-HA produced at dispersion D2, were inserted into subcutaneous pockets in the dorsum of 12-week-old Balb/c mice weighing 25–30 g. Six samples per group and condition were implanted. Six samples of each group were analyzed by micro-CT, histology and immunostaining of CD31 after 2 and 4 weeks of implantation.

### Micro-computed tomography (Micro-CT)

Samples harvested after 2 and 4 weeks, were fixed in 4% (w/v) paraformaldehyde, and were treated by micro-CT before histological analysis. The X-ray microtomographic devices used in this study is a Quantum FX Caliper (Life Sciences, Perkin Elmer, Waltham, MA). The X-ray source was set at 90 V and 160 μA. Sample up to a Field of View of 10 mm diameter and were imaged with a 3D isotropic voxel size of 20 μm. Full 3D high-resolution raw data are obtained by rotating both the X-ray source and the flat panel detector 360° around the sample, with a rotation step of 0.1° (scanning time: 3 min). The corresponding 3,600 image projections were then automatically reconstructed (RigakuSW software, Caliper) into a Dicom stack of 512 files using standard back-projection techniques. The three-dimensional (3D) images of samples were built by stacking 512 cross sections obtained by X-ray microtomography. The resulting 3D files were composed of grey-level images where lowest grey/dark pixels represented empty spaces and highest grey/bright pixels stood for the densest materials. Three-dimensional analyses were performed using eXploreMicroView^®^ software (General Electric Healthcare, Milwaukee, WI). After reconstruction of the region of interest, mineralized volume (MV) and total volume (TV) volume were measured for each group. Six samples were evaluated for each condition at each time point. Results were expressed as average ± standard deviation.

### Histological evaluation

At 2 and 4 weeks post-implantation, samples were retrieved and fixed in 4% (w/v) paraformaldehyde for 24 h at 4°C, then decalcified with Microdec (MM France) during 1 hour under stirring, dehydrated and paraffin-embedded. Transversal sections (7–9 μm thickness) were prepared and treated with Masson Trichrome for mineralized bone and osteoid staining. The sections were analyzed with an Eclipse 80i light microscope (Nikon, Japan). Pictures were captured with a Nanozoomer 2.0HT (Hamamatsu Photonics France) using objective UPS APO 20X NA 0.75 combined to an additional lens 1.75X, leading to a final magnification of 35X. Virtual slides were acquired with a TDI-3CCD camera. Images were analysed using the Nikon software. The whole surface as well as the newly bone surface were quantitated in mm^2^ using the Polygon and Auto Detect functions. Stained slides from two samples per condition were processed for histological analysis, and three sections were fully imaged and analysed per sample and per condition. Results are shown as means with standard deviation per condition, with time of implantation (weeks 2 and 4).

### Immunolabelling of CD31

At 2 and 4 weeks post-implantation, samples were retrieved and fixed in 4% (w/v) paraformaldehyde for 24 h at 4°C, then decalcified with Microdec (MM France) during 1 hour under stirring. Then the samples were dehydrated and embedded in paraffin. Sections (10 μm in thickness) were deparaffinized using toluene, rehydrated in decreasing concentrations of ethanol (100–50%) and finally washed in distilled water. Antigen recovery was performed with proteinase K, diluted at 1/20 within TE buffer (50mM Tris Base, 1mM EDTA, pH 8.0) at 37C for 25 min. Then, endogenous peroxidase was quenched in 3% H_2_O_2_, in PBS, for 5 min. After washing with PBS 0.1M pH7.4, slides were blocked using 2% (w/v) BSA serum in PBS 0.1M pH7.4 for 30 min. For CD31 immunolabeling, incubation was performed overnight with a rabbit polyclonal anti-mice CD31 antibody (dilution 1:100; #100–2284; Novus Biologicals, Littleton, CO, USA) at 4°C. After two washes with PBS 0.1M pH7.4, the slides were incubated with according to the manufacturer’s instruction (Anti-rabbit ABC kit; Vector Labs, Burlingame, CA, USA), and then revealed using Impact DAB solution (Vector Labs, Burlingame, CA, USA). Staining was stopped in distilled water, samples were then counterstained in Mayer’s hematoxylin and washed in running tap water for 10 min. Finally, samples were dehydrated and mounted using Pertex. The number of vessels within the tissue was quantified using NDP view software. The whole surface and the number of vessels were quantified in mm^2^. Stained slides from two samples per condition were processed for immunostaining analysis, and three sections were fully imaged and analysed per sample and per condition. Results are shown as average with standard deviation per condition, with time of implantation (weeks 2 and 4).

### Statistical analysis

All data were expressed as average ± standard deviation (SD). They were analyzed using the t-test. Differences were considered significant when *p*< 0.05 (*). The symbol ** indicates a significant difference with *p*< 0.01; *** a significant difference with *p* < 0.001; ns: non-significant difference.

## Results

### Physicochemical characterization of HA particles

**Fig 1:**
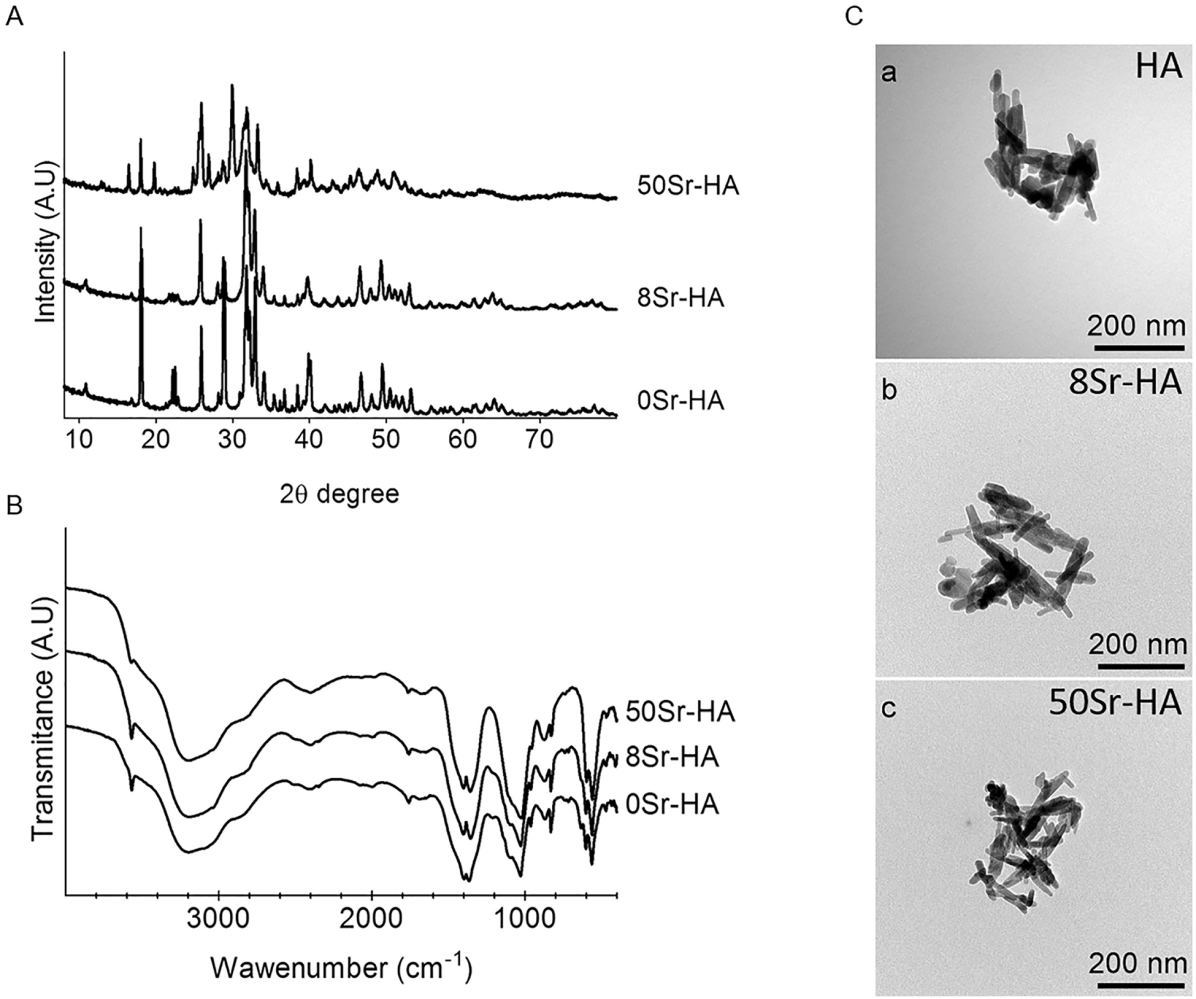
Structural characterization of the synthesized samples of hydroxyapatite. (A) XRD patterns of non-substituted HA samples (HA); HA powders with 8% of Sr-substitution (8Sr-HA); HA powders with 50% of Sr-substitution (50Sr-HA). (B) FTIR spectra of non-substituted HA samples (HA); HA powders with 8% of Sr-substitution (8Sr-HA); HA powders with 50% of Sr-substitution (50Sr-HA). (C) Representative images of HA powders (a: HA, b: 8Sr-HA, c: 50Sr-HA) by Transmission Electron Microscopy (TEM). Scale bars = 100nm.

The X-ray diffraction (XRD) patterns of the powders, precipitated under different experimental conditions, *i*.*e* non-substituted HA (HA), Sr-substitution at 8% (8Sr-HA) and at 50% (50Sr-HA) are shown in Fig 1A. The major diffraction peaks identified for HA are in agreement with the standard JCPDS reference #896438 as a carbonated hydroxyapatite. Major sharp and intense peaks are observed at 2θ values of 25.9°, 31.9°, 32.5° and 32.9°. XRD diagrams of the8Sr-HA powders revealed a similar pattern than those of HA reported in the literature [14, 17], suggesting that 8% of Sr-substitution (8Sr-HA) did not alter, neither the phase composition, nor the crystallinity of the hydroxyapatite. As the Sr-substitution increased to 50% (50Sr-HA), the peak positions were shifted to slightly lower 2-theta values and showed significant broadening.

Representative FTIR spectra in the range of 4000–400 cm^-1^for the synthetized HA and Sr-HA are shown in Fig 1B. Peaks assignments of the three HA powders (HA, 8Sr-HA, 50Sr-HA) and two HA references were reported in Table 2. FTIR analysis displayed specific vibrations for bands present in hydroxyapatite crystals. The major functional groups including hydroxyl, phosphate and carbonate were indicated in Table 2. For HA spectra, the ion stretching vibration around 3500-3600cm^-1^ confirms the presence of a hydroxyl group. Absorption bands characteristic of P-O stretching vibrations are observed at 1029, 962 cm^-1^, while O-P-O bending vibrations can be seen at 563, and 602cm^-1^. Furthermore, peaks associated with carbonate (CO_3_^2−^) were observed at 1364, 1397cm^-1^ and 870cm^-1^. FTIR spectra of 8Sr-HA powders show a similar structure and peak intensity than the HA samples. The corresponding PO4^3-^ peaks for 50Sr-HA shifted to slightly lower wavenumbers (559, 598 cm^−1^) compared to HA powders (563, 602cm^−1^) or to 8Sr-HA powders (562, 601cm^−1^).

**Table 2:** Peak assignments for the different HA powders. Summary of vibrational frequencies observed by FTIR for standard carbonated HA as described by Cox *et al* [17], for non-substituted HA produced by wet precipitation according to Catros *et al* [14], or measured for non-substituted HA, 8Sr-HA and 50Sr-HA synthesized in this work.

Samples	Peak assignment					
	Adsorbed H_2_O	CO_3_^2-^ groups (v3)	PO_4_^3-^ (v3)	PO_4_^3-^ (v1)	CO_3_^2-^ group	PO_4_^3-^ (v4)
Carbonated HA described [17]	2750–3750	1420, 1455	1030, 1090	961	873	604, 600
HA described in [14]	3568	1420	1033	960	868	570, 601
HA	3570	1364, 1397	1029	962	870	563, 602
8Sr-HA	3574	1355, 1401	1027	961	873	562, 601
50Sr-HA	3575	1357, 1401	1023	956	872	559, 598

The structural morphology of the HA particles for all groups was investigated by TEM (Fig 1C). Morphological analyses display aggregates of hydroxyapatite composed of elementary particles of 50–100 nm rod-like crystals. As observed in Fig 1C, Sr-substitution (8Sr-HA and 50Sr-HA) did not alter the shape and the sizes of these particles as well as the formation of micrometric aggregates of these particles.

Dynamic light scattering (DLS) was used to determine the size of the HA samples for all conditions (Table 3). The agglomerated structures of the produced powders impeded the precise measurements of the size and the distribution of the nanoparticles and only confirmed the presence of HA micrometric agglomerates with an average size of 3.2± 0.62 μm to 4.3 ± 0.85 μm, without significant differences between the three groups of HA suspensions (HA; 8SrHA and 50Sr-HA) (Table 3).

**Table 3:** Average agglomerate sizes of HA particles as determined by Dynamic light scattering (DLS). Values shown (in μm) represent the average ± SD of *n* = 3 samples for each condition.

HA powder samples	Average diameter of HA aggregates (μm)
HA	3.26± 0.62
8Sr-HA	4.15± 0.83
50Sr-HA	4.35± 0.85

The amount of Ca and Sr in HA, 8Sr-HA and 50Sr-HA powders was measured by ICP-OES. With increasing Sr-substitution in the hydroxyapatite (8% and 50% of substitution), Table 4 showed an increasing amount of Sr in the HA samples from 2 ± 1 μg / mg of powder (8Sr-HA) to 9.7 ± 3.8 μg / mg of powder (50Sr-HA), and a decreasing content of calcium from 13 ± 8.5 μg / mg of powder (HA), 10 ± 4.7 μg / mg of powder (8Sr-HA) in 5 ± 2.7 μg / mg of powder (50Sr-HA).

**Table 4:** Ca and Sr contents for the HA samples measured by ICP-OES. Powders were preliminary dissolved in 0.5 mL HNO3 and 0.5 mL HCl for 2–3 days before ICP analysis. The resulting solutions were analysed with respect to Ca and Sr concentration with ICP-OES analysis instruments (ICP-OES 720-ES, Varian) calibrated with standard solutions of known concentrations of the elements. Based on the measured concentrations, the concentration of each individual element per suspension was calculated (n = 3). Data are expressed in μg of element / mg of powder (average ± SD).

HA powder samples	Element	Concentration (μg of element / mg of HA powder)
HA	Ca	13 ± 8.5
	Sr	Non detected
8Sr-HA	Ca	10.7± 4.7
	Sr	2 ± 1
50Sr-HA	Ca	5 ± 2.7
	Sr	9.7 ± 4.8

### Effect of the amount of HA dispersed in the 3D matrices on ectopic bone mineralization

**Fig 2:**
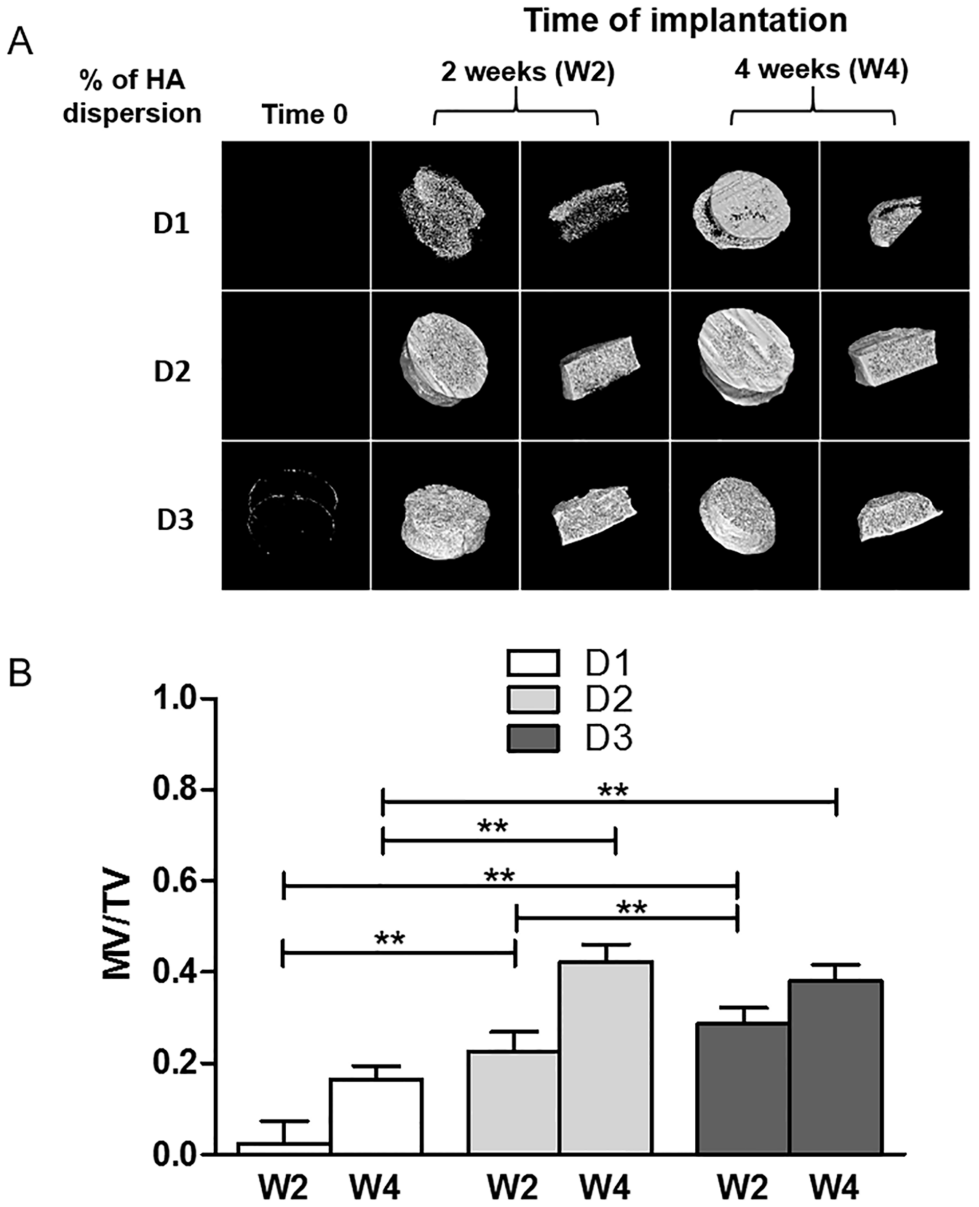
Micro-CT analysis of Matrix HA containing different ratios of HA particles, implanted subcutaneously in mice. (A) Representative Micro-CT images of Matrix-HA supplemented with three different amounts of HA (dispersion 1 (D1), dispersion 2 (D2), and dispersion 3 (D3)), before implantation (Time 0), after 2 weeks (W2) and 4 weeks (W4) of subcutaneous implantation. (B) Quantification of the mineralized volume / total volume (MV/TV) after 2 weeks (W2) and 4 weeks (W4) of subcutaneous implantation. Six samples were evaluated for each condition at each time point (2 and 4weeks). Results were expressed as average ± SD. The symbol ** denotes p<0.01.

Polysaccharide-based matrices (6 mm diameter, 1.5 mm thickness) were first prepared using non-substituted HA particles dispersed in the aqueous polymer solutions at different ratios, D1, D2 and D3, to optimize the amount of the mineral phase in the composite matrix. As reported in Table 1, the dispersion D1, D2 and D3 provide around 14%, 30% and 61% (w/w) of the mineral phase within the matrix, respectively. These three formulations of Matrix-HA were implanted subcutaneously in mice, and explants were analysed after 2 and 4 weeks of implantation by micro-CT. Composite matrices were also imaged before implantation (Time 0). The results in Fig 2A showed that both D1 and D2 of matrix-HA formulations exhibited radiolucent property before implantation (Time 0). Only few spots of mineralized tissue were observed in Matrix-HA supplemented with around 61% of HA (D3). Thereafter, the tissue mineralization increases in all groups with time of implantation. Mineralized structures were observed mainly in the periphery of Matrix-HA produced with D1 ratio after 2 weeks of implantation, compared to those produced with D2 and D3formulations, where mineralized structures were evidenced in the whole explants at week 2.

Quantitative analysis of these images confirmed these qualitative observations (Fig 2B). The mineralization values (ratio of mineralized volume over total volume—MV/TV) of Matrix-HA produced with 14% (D1) and 30% of HA (D2) increase over time. After 2 and 4 weeks of implantation, values for Matrix-HA samples produced with 30% of HA (D2formulation) were significantly higher than Matrix-HA produced with 14% of HA (D1 formulation) (p<0.01). When evaluating MV/TV for the Matrix HA produced with around 61% of HA (D3formulation), there were no statistically significant differences between the two time points of implantation (2 and 4 weeks). There are no significant differences of MV/TV values between the Matrix-HA produced at D2 and a Matrix-HA produced at D3, after 4 weeks of implantation. We then selected the ratio of HA to polymer of 30% (D2 dispersion) for the further synthesis of Matrix-HA doped with strontium.

### Synthesis and physicochemical characterizations of the Matrix-HA supplemented with strontium

**Fig 3:**
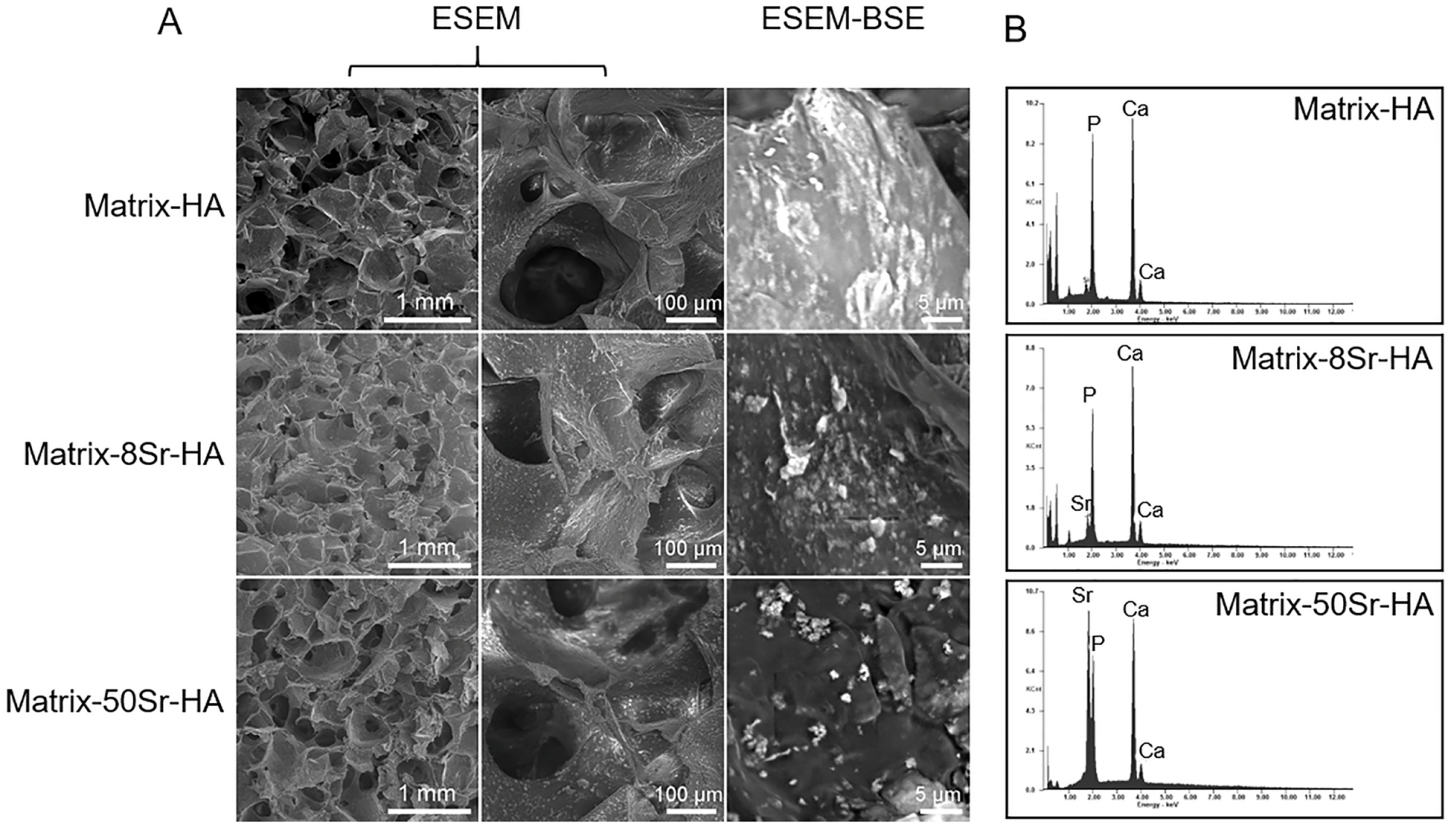
Characterization of the Matrix-HA. (A) Environmental scanning electron microscopy (ESEM) and back scattering electron microscopy (ESEM-BSE) images showing the presence of the macroporous structure of the 3D matrices and the presence of numerous HA particles (indicated by white arrows). Sale bars = 0.5 mm and 100 μm for ESEM; and 5 μm for ESEM-BSE (B) Energy Dispersive X-ray spectroscopy (EDX) spectra of Matrix-HA, Matrix-8Sr-HA, and Matrix-50Sr-HA.

Three types of Matrix-HA were produced with three formulations of substituted HA powders at dispersion D2, *i*.*e* non-substituted HA (named Matrix-HA), 8% of Sr-substitution (named Matrix-8Sr-HA) and 50% of Sr-substitution (named Matrix-50Sr-HA). ICP-OES used to determine the strontium and calcium content in matrices confirmed that the Sr content increased with Sr-substitution (34.9 ± 2.9 ng of Sr per Matrix-8Sr-HA; and 198.8 ± 4.4 ng of Sr per Matrix-50Sr-HA). ESEM images of hydrated matrices (Fig 3A) showed similar macroporous structures whatever the composition, with a pore size from 10 to 400 μm. ESEM-BSE (Fig 3A) revealed the presence of HA agglomerates dispersed within the 3D structure. EDX analyses performed at different locations of the composite scaffolds confirmed the presence of these particles and a homogenous distribution of HA particles within the polysaccharides, whatever the Sr-substitution. The EDX spectra of the Matrix-HA and Matrix-Sr-Ha samples (Fig 3B) evidenced the presence of calcium and phosphorus. Strontium was detected in Matrix-8Sr-HA and peak was higher in Matrix-50Sr-HA. The average Ca/P and Ca+Sr/P ratios were calculated from three EDX measurements performed at different samples locations. The Ca/P ratio of the Matrix-HA samples was 1.80 ± 0.1. The ratio of Ca+Sr/P was 2.18 ± 0.20 and 2.90 ± 0.70 for Matrix-HA-8Sr-HA and Matrix-HA-50Sr-HA, respectively.

### Cytotoxicity evaluation of the Matrix-HA supplemented with strontium

**Fig 4:**
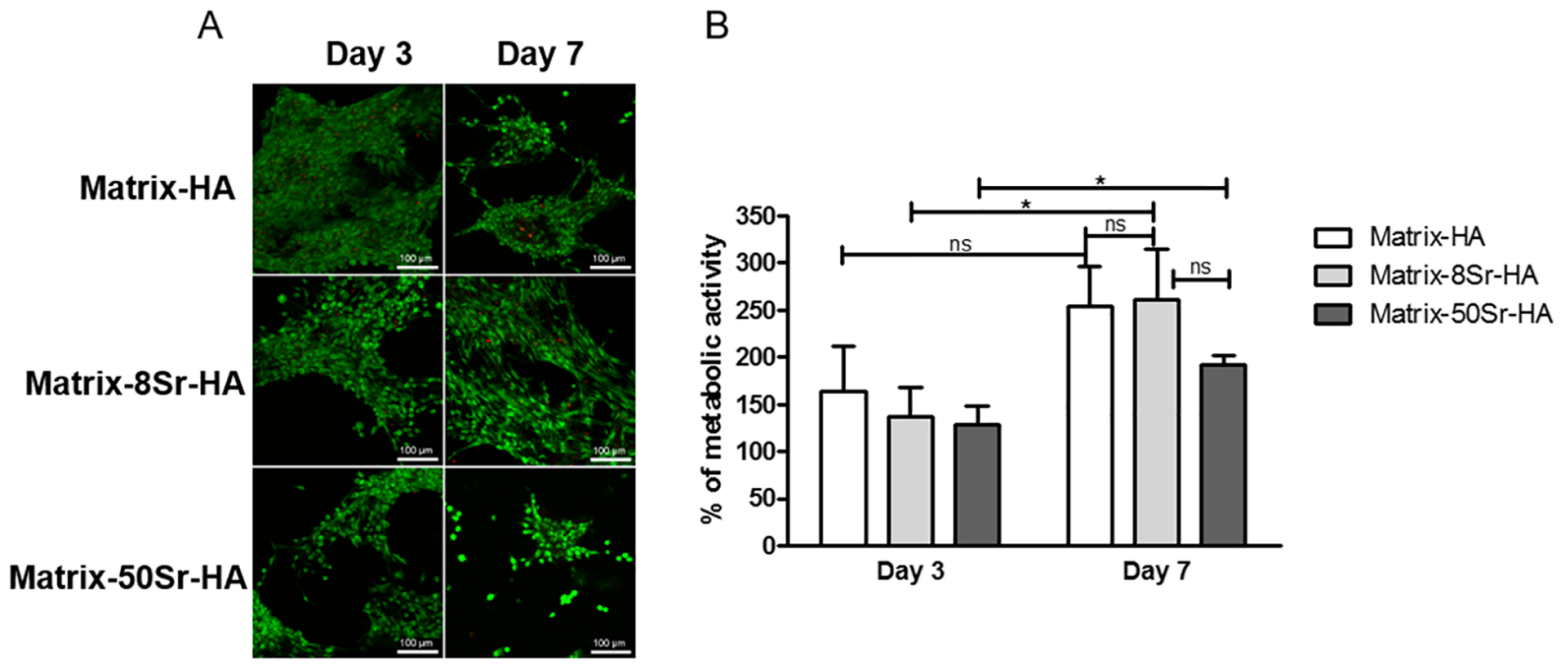
Effect of the various Matrix-HA doped or not with strontium on MSCs viability. (A) LIVE/DEAD^®^ assays after 3 and 7 days of culture of MSCs on the three different matrices (Matrix-HA, Matrix-8Sr-HA and Matrix-50Sr-HA). Scale bars = 50 μm. (B) Alamar Blue assay after 3 and 7 days of culture of MSCs on the three different matrices (Matrix-HA, Matrix-8Sr-HA and Matrix-50Sr-HA). Data were expressed in % of metabolic activity normalized to day 1 (100%) for each condition (average ± SD). The symbol * denotes *p*<0.05 and ns, “non-significant”.

After 3 and 7 days of culture, LIVE/DEAD^®^ assay (Fig 4A) revealed that all matrices supplemented with HA particles doped or not with strontium had no obvious cytotoxicity to MSCs over time of culture, whatever the rate of Sr-substitution of HA. Alamar blue assay (Fig 4B) confirmed that cells cultured on these 3D scaffolds maintain their metabolic activity with time of culture. On the third day of culture, there were no statistical significant differences between metabolic activity of MSCs cultured on the various matrices. With increasing the culture duration (Day7), MSCs cultured on all scaffolds, with or without strontium, exhibited significant higher metabolic activity compared to day 3. At day 7, the metabolic activity of MSCs cultured in Matrix-HA and Matrix-Sr-HA was comparable. However, there is a significant difference of metabolic activity of MSCs cultured on Matrix-8Sr-HA and Matrix-50Sr-HA.

### MSCs differentiation on the Matrix-HA supplemented with strontium

**Fig 5:**
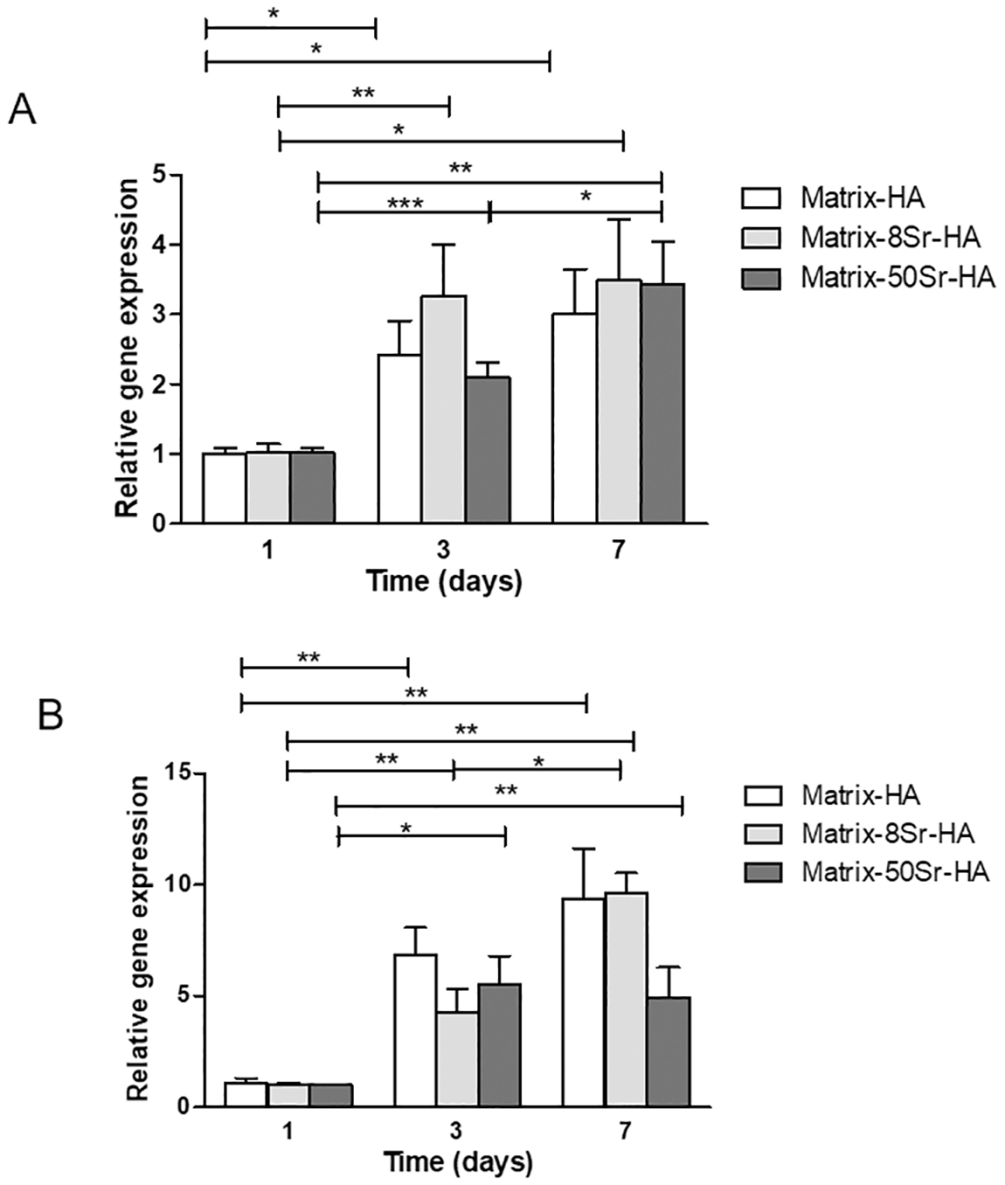
mRNA expression of early and late osteoblastic gene in MSCs cultured on the three different matrices. Runx 2 (A) and OPN (B) expression were quantified by qPCR after 1, 3 and 7 days of hMSCs cultured on Matrix-HA, Matrix-8Sr-HA and Matrix-50Sr-HA. Three separate experiments were performed and each assay was done in duplicate. Data were expressed in relative expression normalized to P0 expression, compared to day 1. Runx2 (A) and OPN (B) gene expression were set as “1.0” at Day 1 for the three scaffolds. Results were expressed as average ± SD. The symbol * denotes *p*< 0.05; ** denotes *p*<0.01 and *** indicates a significant difference with *p*< 0.001.

The expression levels of osteogenesis related genes including Runx2 as early gene (Fig 5A and Table 5), and osteopontin (OPN) as a late osteoblastic gene (Fig 5B and Table 5) were examined by qPCR after culturing in presence of the different matrices without osteogenic additives.

**Table 5:** mRNA expression of Runx2 and osteopontin (OPN) gene in MSCs cultured on the three different matrices. Runx 2 and OPN expression were quantified by qPCR after 3 and 7 days of MSCs cultured on Matrix-HA, Matrix-8Sr-HA and Matrix-50Sr-HA. Three separate experiments were performed and each assay was done in duplicate. Data were expressed in relative expression normalized to P0 expression, compared to Matrix-HA for each time. Gene expression were set as “1.0” for each day (Day 3 and Day 7) for Matrix-HA. Results were expressed as average ± SD.

		*Runx2*	*OPN*
		QR average ± SD	QR average ± SD
**Day 3**	**Matrix-HA**	1.0 ± 0.2	1.0 ± 0.1
	**Matrix-8Sr-HA**	1.0 ± 0.1	7.8 ± 2.2 *
	**Matrix-50Sr-HA**	1.3 ± 0.2 *	4.1 ± 1.4 **
**Day 7**	**Matrix-HA**	1.0 ± 0	1.0 ± 0
	**Matrix-8Sr-HA**	0.8 ± 0.1	1.0 ± 0.5
	**Matrix-50Sr-HA**	1.0± 0.1	1.1 ± 0.3

The symbol * denotes *p*< 0.05 and ** denotes *p*<0.01.

Relative expression of OPN was expressed firstly with time of implantation (Fig 5) for each type of scaffolds and data were normalized to Day 1 for each matrix. OPN and Runx2 gene expression were set “1.0” at Day 1 for the three scaffolds. Fig 5A and 5B revealed that the presence of Sr within the matrices did not affect the expression of Runx2 and OPN over time of the 3D cell culture, whatever the level of Sr-substitution. The relative expression of Runx2 in MSC cultured on the three scaffolds, increases significantly from D1 to D7 (Fig 5A). For cells cultured into Matrix-HA and Matrix-8Sr-HA samples, the expression of OPN increases significantly from Day 1 to Day 7 (Fig 5B). For the Matrix-50Sr-HA group, the expression of OPN in MSCs reached a plateau at Day 3 (Fig 5B). At Day 7, the relative expression of OPN, normalized to Day 1, did not change compared to Day 3 (Fig 5B).

In Table 5, the relative expression of OPN was normalized for each time point (Day 3 and Day 7) to Matrix-HA deprived of strontium. We have evaluated here the effect of strontium (Sr) contained in the composite matrix on the expression of Runx2 and OPN at each time-point of culture of hMSCs.

This Table indicates that the presence of strontium within the matrix, i.e Matrix-8Sr-HA and Matrix-50Sr-HA does not modify Runx2 expression, whatever the time of culture (3 or 7 days), but significantly stimulates the expression of OPN only after 3 days of culture, compared to the composite matrix deprived of Sr (Matrix-HA) at the same time (Day 3). However, this is a transient effect. Strontium has a short-term effect on OPN gene expression, and this effect was not maintained after 7 days of culture.

### In vivo evaluation of the Matrix-HA supplemented with strontium implanted in ectopic site

**Fig 6:**
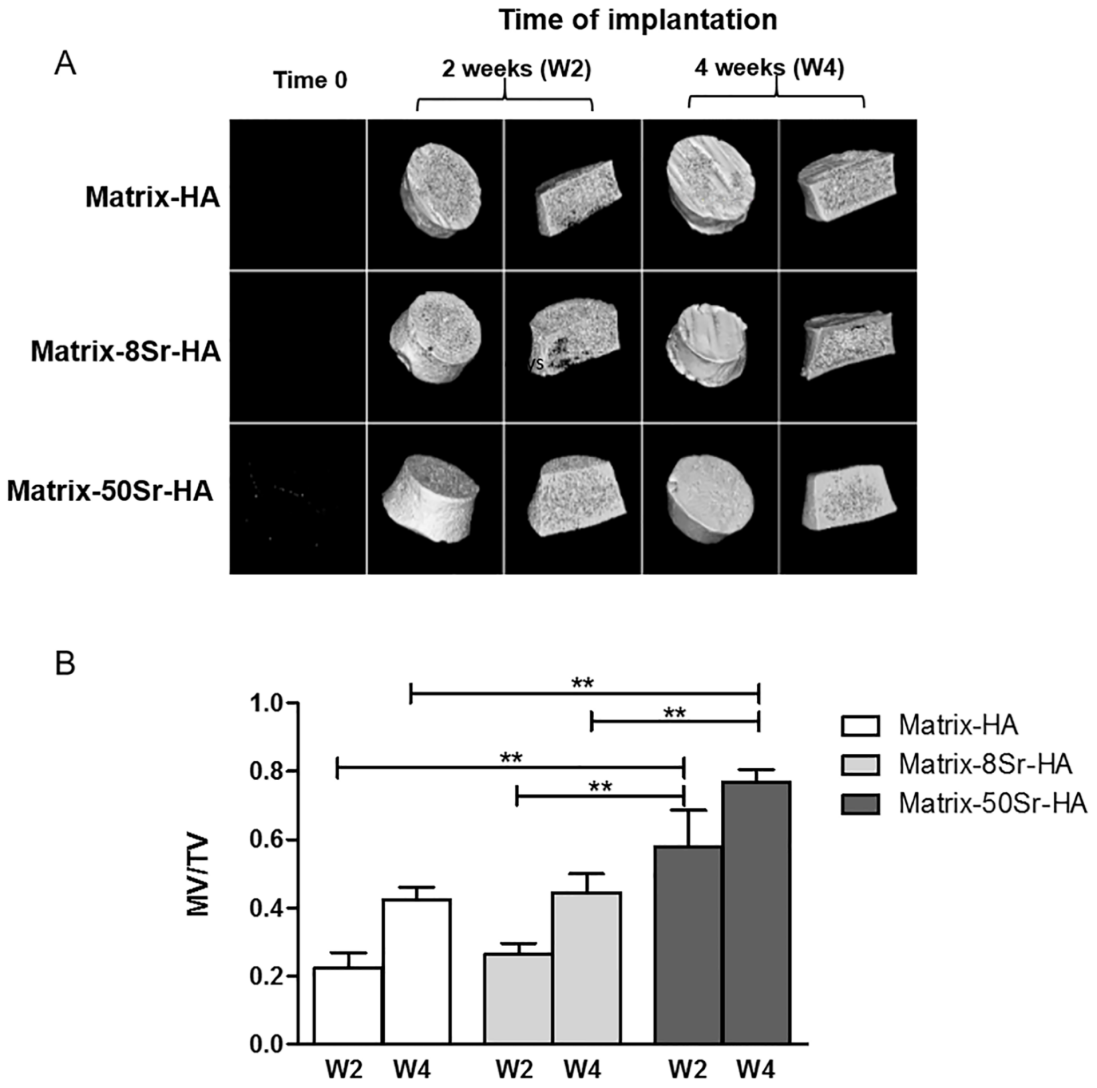
*In vivo* evaluation of the Matrix-HA supplemented with strontium implanted subcutaneously in mice. (A) Representative micro-CT images of Matrix-HA, Matrix-8Sr-HA and Matrix-50Sr-HA, before the implantation (Time 0), and after 2 (W2), and 4 weeks (W4) of subcutaneous implantation. (B) Quantification of the mineralized volume / total volume (MV/TV) after 2 weeks (W2) and 4 weeks (W4) of subcutaneous implantation. Six samples were evaluated for each condition at each time points 2 weeks and 4weeks (W2, W4). Results were expressed as average ± SD. The symbol ** denotes *p*<0.01.

**Fig 7:**
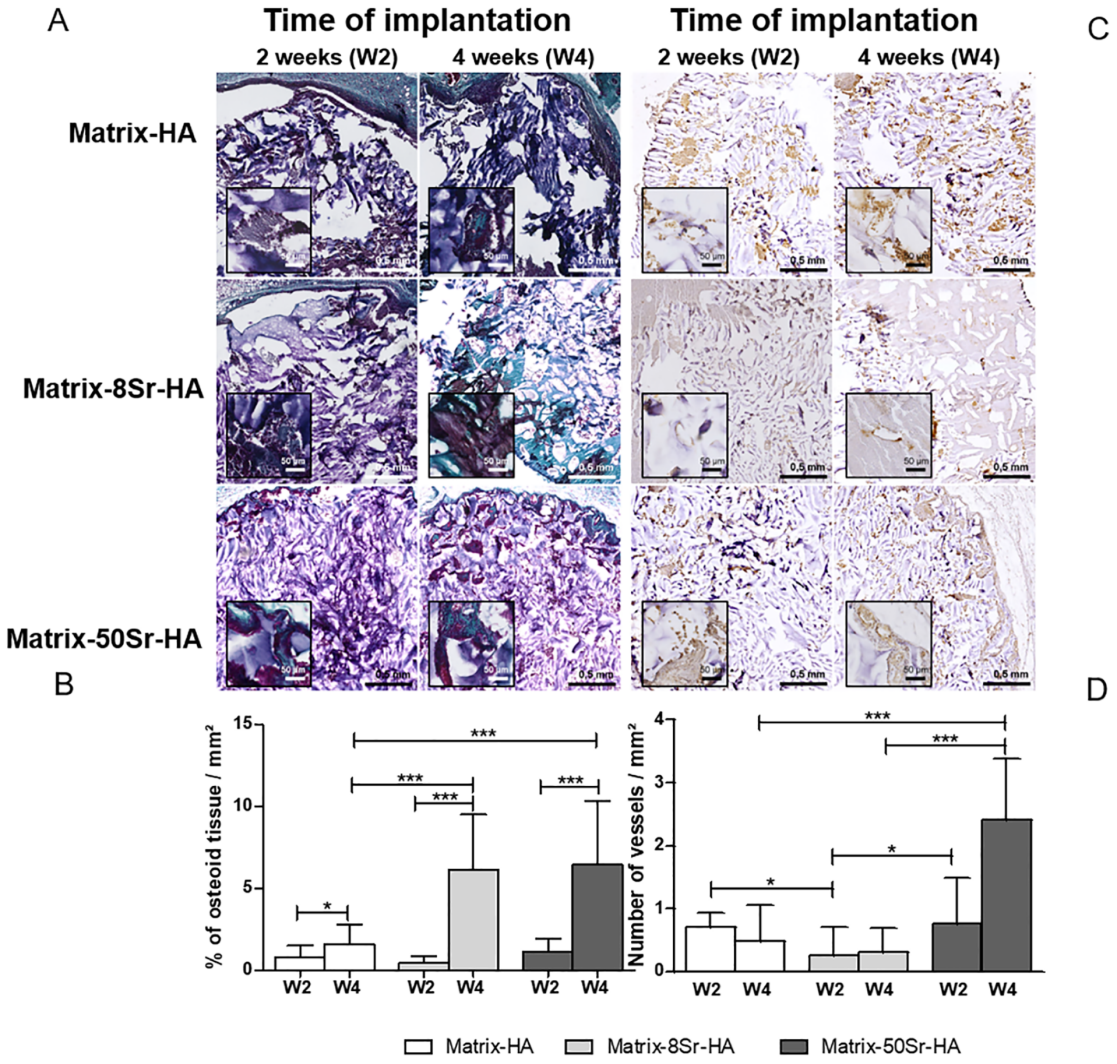
Histological and immunochemistry analysis of the newly formed tissue within the Matrix-HA supplemented with strontium, implanted subcutaneously in mice. (A) Masson’s Trichrome staining of the newly tissue formed within the three matrices after 2 and 4 weeks of subcutaneous implantation. (B) Quantitative analysis: images were analysed using the Nikon software. The whole surface as well as the newly bone surface were quantified in mm^2^. Stained slides from 2 samples per condition were processed for histological analysis, 3 sections were fully imaged and analysed per sample and per condition. Results were expressed as average ± SD per group of matrix, with time of implantation (2 and 4 weeks: W2, W4). The symbols *; **; and *** denote *p*<0.05; *p*<0.01 or *p*<0.001, respectively. (C) CD31 immunostaining of the newly formed tissue within the three matrices, Matrix-HA, Matrix-8Sr-HA and Matrix-50Sr-HA. (D) Quantification of the number of vessels within the tissue was performed by using NDP view software. The whole surface as well as the number of vessels were quantitated in mm^2^. Stained slides from 2 samples per condition were processed for immunostaining analysis, and 3 sections were fully imaged and analysed per sample and per group of matrix. Results were expressed as average ± SD per group of matrix, with time of implantation (2 and 4weeks: W2, W4). The symbols *; **; and *** indicate *p*<0.05; *p*<0.01 or *p*<0.001, respectively.

Tissue formed after 2 and 4 weeks of subcutaneous implantation in mice of Matrix-HA, Matrix-8Sr-HA and Matrix-50Sr-HA, was analysed by micro-CT (Fig 6), histology (Fig 7A and 7B) and by immunostaining of CD31 for the blood vessel staining (Fig 7C and 7D). As shown in Fig 6A, micro-CT images showed that all the tested matrices exhibited radiolucent properties before implantation (Time 0) and that mineralized tissues were observed from two weeks of implantation for the three groups of matrices. Quantitative analysis of micro-CT (Fig 6B) revealed an increase of MV/TV over time of implantation with all matrices. However, the amount of mineralized tissues in the Matrix-50Sr-HA samples was significantly higher than those of the Matrix-HA and Matrix-8Sr-HA samples, for both time points (2 and 4 weeks).

The Trichrome Masson’s staining of the newly formed tissue (Fig 7A) and the quantification of the osteoid tissues (Fig 7B) confirmed that the supplementation of strontium in the matrices, whatever the dose, has stimulated the osteoid tissue formation after 4 weeks of implantation. The formation of vessels was also analyzed by CD31 immunostaining (Fig 7C). only few vessels were observed in the Matrix-HA and Matrix-8Sr-HA groups in comparison to Matrix-50Sr-HA explants, mainly after 4 weeks of implantations (Fig 7D), suggesting a positive effect of strontium on angiogenesis.

## Discussion

Our previous studies demontrates the ability of polysaccharide-based matrices supplemented with 14% of HA particles (here, corresponding to D1 as ratio of HA particles) to promote mineralization and osteoid tissue formation, in two different ectopic sites (subcutaneous and intramuscular), and in two different experimental models, in mice and goat [35]. Here in this work, we have generated new formulations of polysaccharide-based matrix. Thus, we have optimized the ratio of HA particles within the matrix and compare the efficacy of three ratios of dispersion of HA particles. An increase of HA particles to 30% in the matrices (D2 formulation) improved their bioactive properties. The *in vivo* data also showed that partial substitution of Sr for Ca in hydroxyapatite enhanced tissue mineralization, osteoid and new blood vessel formation. We discreminate here an optimal composition of the Sr-doped Matrix-HA for promoting bone formation and vascularization in an non-osseous site.

In this study, two different Sr-substituted hydroxyapatite powders (8Sr-HA and 50Sr-HA) were produced and compared for their structure and composition with non-doped HA. XRD and FTIR analysis of HA powder confirmed the presence of a carbonated hydroxyapaptite. As described in the literature, the composition of the HA powder changes with strontium incorporation and the physical properties may be affected as a result [17, 34]. In this study, a change in composition were confirmed by ICP-OES with an increase of strontium content and decrease of calcium. The XRD results did not show a corresponding change in the cristallinity of 8Sr-HA. However, slight deviations in XRD peaks with 50Sr-HA, with significant broadening may indicate a change of crystal structure as described by others [39]. On the other hand, the corresponding FTIR spectra highlighted that, as the Sr content of these powders increased, there was significant peak broadening for the PO4^3-^ peaks, as well as a shift to slightly lower wave numbers which is indicative of Sr incorporation in the HA lattice [28]. Both the Sr-substituted materials are also becoming more dehydroxylated with increasing Sr content with, the OH- band in the 50Sr-HA FTIR spectra around 3575 cm^-1^, becoming much less prominent compared to HA FTIR spectra. Sr-substitution for Ca in the HA lattice also results in an increase in both CO_3_^2-^ and HPO4^2-^ due to the increased lattice strain caused by the Sr larger ions [40].

The structural morphology of the three HA powders analysed by TEM and DLS gave the direct information about the shape and size of the synthesized samples. Both methods showed the formation of non-uniform large aggregates of nanometer rod-like crystals without any significant differences in shape or sizes of these agglomerates with Sr-substitution. The levels of Sr-substitution in our study did not influence the particle morphology, as previously described [34]. The presence of nano-sized particles (less than 100 nm) was undetectable by DLS, whatever the conditions of treatment of these HA suspensions even after the sonication. Aggregates of HA particles were maintained, once dispersed in the polysaccharide matrices as showed by ESEM and ESEM-BSE analysis. Aggregates were homogeneously dispersed in the whole matrices as demonstrated by EDX analysis. It can be hypothesized that the dispersed particles can act as nucleation sites for the mineralization at the particle interface, which, in turn, leads to enhancement of the bioactive properties of the composite scaffolds

Herein, polysaccharide-based composite scaffolds were produced with increasing amount of HA. In this work, three concentrations of micro-sized hydroxyapatites aggregates were used to produce these biocomposites. Our previous work was performed using Matrix-HA supplemented with 14% of HA. Here, we improved significantly the ability of the scaffold to promote tissue mineralization by doubling the amount of HA (30%) in the formulation. Interestingly, no significant effect on tissue mineralization evidenced by micro-CT was observed by an additional increase of HA at 61%. Higher content of HA particles could result in more HA aggregation and a less uniform distribution of these agglomerates with a loss of bioactivity. These data are in accordance with the literature. Boissard *et al* also showed that at higher filler contents the HA particles aggregate may hinder the mobility of the polymer matrix [41]. The same authors demonstrate that the micro-CT scans of scaffolds showed that scaffold pore size and porosity decreased with an increase in HA content. Consequently, several physical parameters are affected by an increase of HA that could affect with the implantation time the overall matrix properties such as in-and-out diffusion coefficients, degradation rates, cellular infiltration and differentiation.

In addition, the Matrix-HA with the higher HA content (61%) started to be radiopaque as showed in Fig 2A. Indeed, from a clinical point of view, the radiological follow-up of the samples implanted can be used to observe from the beginning of the surgery the extent of mineralization inside a grafted site with time, as a good indicator of the success of the bone graft.

The second step of our study was to incorporate strontium into the biocomposites. Bone typically contains 70% inorganic calcium phosphate and different trace elements, such as strontium, a non-essential element, which is about 0.035% of its calcium content in the skeleton system. The importance of this element in natural bone health and ingrowth is well documented and the effect of a wide range of concentrations of Sr on both *in vitro* and *in vivo* bone formation and mineralization was largely investigated. It could also exert a positive effect on endothelial cells and angiogenesis process. Since Sr acts as a dual agent on bone formation and resorption, the optimal content of Sr remains the subject of investigation. As described in the literature, the levels of Sr-substitution in HA or in other ceramics vary from 1% to 100% [34]. A complex dose-dependent effect of this element was found according to the mode of HA synthesis and/or to the nature of the polymer in which it was incorporated (PLA, PLGA, collagen, or chitosan). Herein, different formulations of Matrix-HA containing 30% of Sr-HA substituted at two different ratios (8 and 50%) were designed (Matrix-HA, Matrix-8Sr-HA and Matrix-50-Sr-HA). As expected, the Sr content within the Matrix-Sr-HA, measured by ICP-OES, was increased with increasing Sr-substitution in HA from 34.9 ± 2.9 ng to 198.8 ± 4.4 ng per matrix (6 mm diameter, 3 mm thickness). EDX spectra of the three matrices revealed an increase of Ca+Sr/P ratio with increasing Sr-substitution suggesting that, more Sr incorporation in HA might lead to crystal change.

In addition, as described in the literature, high concentration of strontium also could to be toxic for cells [42]. However, our *in vitro* studies did not reveal any cytotoxicity of the three matrices, including for Matrix-50-Sr-HA. We were also able to demonstrate that all matrices sustained cell proliferation of MSCs up to 7 days in culture.

Extensive literature has confirmed that Sr-containing materials can stimulate the differentiation of MSCs or other osteoblastic cell lines [29, 43]. Here, we also showed that Sr-HA doped-matrices allowed the differentiation of MSCs to the osteoblastic lineage toward the expression of specific genes (Runx2 and OPN). However, the comparison of relative expression of these genes between the Matrix-HA and the Matrix-Sr-HA group at each time point (Day 3 and Day 7) (Table 5), revealed only a significant effect of strontium at day 3, not maintained at day 7 of culture, especially for OPN expression, remarkably increased at day 3. The quantification of the corresponding protein by ELISA should allow to us confirm the role of Sr on osteoblast differentiation.

The osteoconductive properties of different Sr-doped biocomposites have been investigate din numerous bone defects [44–47]. It has been reported that the implantation of Sr-containing HA materials promotes bone repair in both normal [33] and ovariectomized animals [23, 48], which evidence the role of strontium to enhance bone fracture healing. However, none of these works showed that Sr-containing composites give rise to inductive bone formation in ectopic sites. Recently, Luo *et al*. [49]demonstrated that after heterotopic implantation with the combination of rhBMP-2, bone formation on strontium-containing apatite/poly lactide strontium containing composites was enhanced. The results of this study further address the possible synergic effects of rhBMP-2 and strontium influence in bone formation. Here, our objective was to study the effect of matrices containing Sr (Matrix-8Sr-HA and Matrix-50Sr-HA), deprived of osteoinductive factors, on ectopic bone tissue mineralization after 2 and 4 weeks. The nature of the tissue was analyzed by micro-CT, by histology for the osteoid tissue staining and immunohistochemistry for the quantification of the newly formed blood vessels. Micro-CT showed that matrices supplemented with 50Sr-HA particles were capable of influencing the *in vivo* mineralization process as early as 15 days after subcutaneous implantation in mice. A higher stimulation of tissue mineralization was obtained after 4 weeks with these matrices, where the number of blood vessels in the newly formed tissue also appeared to be higher. Quantification of osteoid formation confirmed that both Sr-doped matrices (Matrix-8Sr-HA and Matrix-50Sr-HA) significantly increased osteoid formation after 4 weeks, compared to non-doped matrix. The overall tissue bone formation was modest, which might be attributed to the short time for bone formation (4 weeks). Furthermore, the Matrix-8Sr-HA did not stimulate blood invasion at the same time point (4 weeks), while it increased osteoid formation. This could be explained by a difference of diffusion of nutrients in materials containing higher amount of Sr that favors blood invasion and angiogenesis. Indeed, it has been reported that materials containing Sr exhibited higher nutrient distribution and permeability than the materials deprived of Sr [46].

Tissue mineralization and osteoid formation may also be controlled by other biological events than vascularization. The release of inorganic ions may also help the formation of a biological carbonated apatite layer that may also control stem cell differentiation. We thus suggested that these strontium-releasing composites provide a local ion-rich (*i*.*e*., Sr^2+^as well as Ca^2+^ and PO4^3−^) environment that favors osteogenic differentiation and *in vivo* bone formation. The strontium release with degradation of these composite materials is now under progress to elucidate the mechanism involved.

## Conclusion

The results of this study show that optimal substitution levels of Sr and apatite content in this 3D polysaccharide matrix provide a suitable microenvironment for allowing osteogenic differentiation of human MSCs *in vitro*. The supplementation of Sr in these matrices leads to enhance tissue mineralization and bone formation in a mice ectopic model. The development of these biodegradable osteoinductive matrices seems promising for use in bone regeneration. Further preclinical studies are now needed to demonstrate the safety and efficacy of these composite materials in large bone defects.
